# Biostimulation effect of platelet-rich fibrin augmented with decellularized bovine pericardium on full-thickness cutaneous wound healing in Donkeys (*Equus asinus*)

**DOI:** 10.1186/s12917-023-03733-x

**Published:** 2023-09-20

**Authors:** Mohammed Albahrawy, Khaled Abouelnasr, Esam Mosbah, Adel Zaghloul, Marwa Abass

**Affiliations:** Department of Surgery, Anesthesiology, and Radiology, Faculty of Veterinary Medicine, Mansoura University, Mansoura, 35516 Egypt

**Keywords:** Platelet-rich fibrin, Donkeys, Bovine pericardium, Wounds

## Abstract

**Aim:**

The current research aimed to evaluate the potential effect of adding platelet-rich fibrin (PRF) to the decellularized bovine pericardium (DBP) on the distal limb of donkeys’ full-thickness cutaneous wounds healing (Equus asinus).

**Materials and Methods:**

Healthy male donkeys (n = 12) were used in this study. Under general anesthesia, 6 cm2 full-thickness incisions were made on the middle dorsolateral surface of both forelimbs’ metacarpi. The left forelimbs were control wounds, while the right wounds were treated with PRF/DBP. Control wounds were bandaged with a standard dressing after saline irrigation and were evaluated at days 4, 7, 10, 13, 16, 19, 22, 25, and 28 post-wounding. PRF/DBP-treated wounds were dressed with a combination of PRF/DBP at the first, second, and third weeks post-wounding. Clinical and histopathological examinations of the wounds were performed to assess the healing process. Additionally, the immunohistochemical evaluation and gene expression profiles of myofibroblastic and angiogenic genes (transforming growth factor-β1, vascular endothelial growth factor-A, fibroblast growth factor 7 (FGF-7), and collagen type 3α1) were analyzed.

**Results:**

PRF/DBP wounds had a significantly faster healing process (61.3 ± 2.6 days) than control wounds (90.3 ± 1.4 days) (p < 0.05). The immunohistochemical examination and gene expression profile revealed significant enrichment in PRF/DBP wounds compared to control wounds.

**Conclusion:**

PRF/DBP dressing can be considered a natural and cost-effective biomaterial for enhancing the recovery of donkeys’ distal limb injuries.

**Authors**:

Phone numbers: +201,015,217,659.

## Introduction

Distal limb wounds in equines are prevalent and display more than 60% of all wounds. Second-intension wound healing is the best route of choice in many equine wounds, especially in cases of extreme contamination, excessive skin tension, massive tissue loss, the undue time elapsed since the injury, or as a result of wound dehisces post prevents the first intension wound healing [1, 2]. Ideal wound healing requires exuberant granulation tissue avoidance, accelerated epithelialization, and increased contraction rate [3].

Wound healing in the horse is more complicated and significantly slow, with a less favorable prognosis compared to ponies and donkeys [4]. During wound treatment, a rapid healing process with minimum scar tissue and maximum function is the goal. Many topical wound medications were used for the enhanced wound-healing process in both animals [5].

Over the past years, the ingredients of the biological scaffolds have been extensively utilized to manage PRF as a single fibrin membrane of platelet and immune concentrates aggregating all blood parameters, which is important for decreasing the postoperative inflammatory process as well as promoting immunity and healing. Furthermore, PRF has six primary angiogenesis soluble factors, including fibroblast growth factor basic (FGFb), transforming growth factor-β1, angiopoietin, platelet-derived growth factor (PDGF), and vascular endothelial growth factor (VEGF). Therefore, it contributes to the process of angiogenesis and makes it an efficient therapy for tissue repair [6–8].

Because of the gradual growth factor release over 14 days in combination with fibrin net disintegration following PRF application, PRF is regarded as a suitable medium for cell proliferation, migration, and differentiation [9]. In veterinary medicine, PRF’s impact on cutaneous wound healing has been examined in equine’s distal limb wounds [8, 10], bucks’ chronic open wounds [11], tenorrhaphy in sheep [12], and dogs [13].

Biologic scaffolds derived from many organs and decellularized tissues have been efficiently implemented, and utilized in pre-clinical animal studies [14] as well as human clinical practices [15].

Bovine, equine, and porcine pericardia have been used both clinically and experimentally for many years. Human and bovine pericardia have been widely used to repair congenital and acquired myocardia and skin defects [16–18]. The decellularization of the bovine pericardium reduced the propensity for calcification in vivo. In addition, following decellularization and sterilization of the pericardium with peracetic acid to reduce the level of glutaraldehyde used to preserve the tissue and generate a tissue with appropriate biological and bio-mechanical properties for preparing cardiovascular patches and cardiac valve prostheses [19]. In addition, [14, 18] reported that the bovine pericardial membrane contributes to promoting abdominal hernial repair in rats as well as skin healing injuries in rabbits [20, 21].

This research aimed to study the potential use of the combined PRF and decellularization of bovine pericardium as a successful dressing route to promote cutaneous healing of donkeys’ distal limbs.

## Results

### Clinical and macroscopic results

In control wounds, the wound boundaries exhibited minor edematous swelling on the third day, accompanied by minor bleeding during the initial bandage change. In addition, the animals exhibited a pain response, as shown by the mild finger pressure on the incision edges, whereas, on the sixth postoperative day, most wounds exhibited a significant reduction in edema. The skin’s sensitivity progressively diminished until it vanished by the end of the second week. In the first week, there was a discernible expansion in wound size (10.2 ± 0.3 cm2), resulting in a delay in wound shrinkage (-22.7 ± 4.6%) (Table 1). The average time for wounds to heal was 86.3 ± 3.1 days.

**Table 1:** Mean values and standard deviations of wound size (cm^2^), wound contraction (%), Epithelization (%) and granulation tissue formation in control and PRF/DBP treated groups during skin wound healing of distal limbs in donkeys

Criteria	Groups	Time post treatment (week)					
		1st	3rd	5th	7th	9th	10th
wound size (cm^2^)	Control	10.2 ± 0.3^a^	8.5 ± 0.08^a^	5.4 ± 0.3^a^	4.7 ± 0.2^a^	4 ± 0.3^a^	3.8 ± 0.2^a^
	PRF/DBP	6.3 ± 0.06^b^	4.2 ± 0.12^b^	2.1 ± 0.1^b^	1.3 ± 0.1^b^	0.7 ± 0.2^b^	0.4 ± 0.1^b^
Wound contraction (%)	Control	-22.7 ± 4.6^a^	10.7 ± 1.8^a^	25.3 ± 4.8^a^	40 ± 2.8^a^	49.5 ± 5.1^a^	55.3 ± 3.5^a^
	PRF/DBP	-5 ± 1^b^	30.7 ± 2^b^	65.7 ± 1.8^b^	78.7 ± 1.9^b^	87.6 ± 1.8^b^	93.6 ± 2^b^
Epithelization (%)	Control	0.00 ± 0.00	8.6 ± 1^a^	30.7 ± 1.8^a^	43.7 ± 1.9^a^	55.8 ± 1.9^a^	60.7 ± 1.8^a^
	PRF/DBP	0.00 ± 0.00	34.6 ± 1.8^b^	69.7 ± 1.8^b^	83.6 ± 1.9^b^	89.9 ± 1.5^b^	94.4 ± 1.6^b^
Wound healing (%)	Control	-24.7 ± 4.6^a^	12.8 ± 1.6^a^	28.6 ± 1.8^a^	45.7 ± 1.6^a^	60.5 ± 1.8^a^	70.7 ± 1.7^a^
	PRF/DBP	-9 ± 1^b^	40.6 ± 1.9^b^	58.7 ± 1.8^b^	79.6 ± 1.9^b^	94.6 ± 4.5^b^	98.8 ± 2.5^b^
Granulation tissue formation score	Control	0.5 ± 0.8^a^	1.8 ± 1.2^a^	1.2 ± 0.75^a^	0.8 ± 0.75^a^	0.8 ± 0.75^a^	0.35 ± 0.01^a^
	PRF/DBP	0.2 ± 0.4^b^	0.5 ± 0.54^b^	0.5 ± 0.54^b^	0.5 ± 0.54^b^	0.3 ± 0.5^b^	0.00 ± 0.00^b^

Means with different superscript letters in the same column are significantly different at p < 0.05

Exudation from control wounds was sanguineous (reddish, watery, and thin) with an excess volume and a normal odor during the first three days. Other wounds demonstrated serosanguinous exudation (pink, watery, and thin). In all control wounds, the exudate became serous (yellowish, watery, and thin) and slight in quantity, with a normal odor throughout the ensuing five days, and then completely vanished on the tenth day postoperatively.

**Fig. 1:**
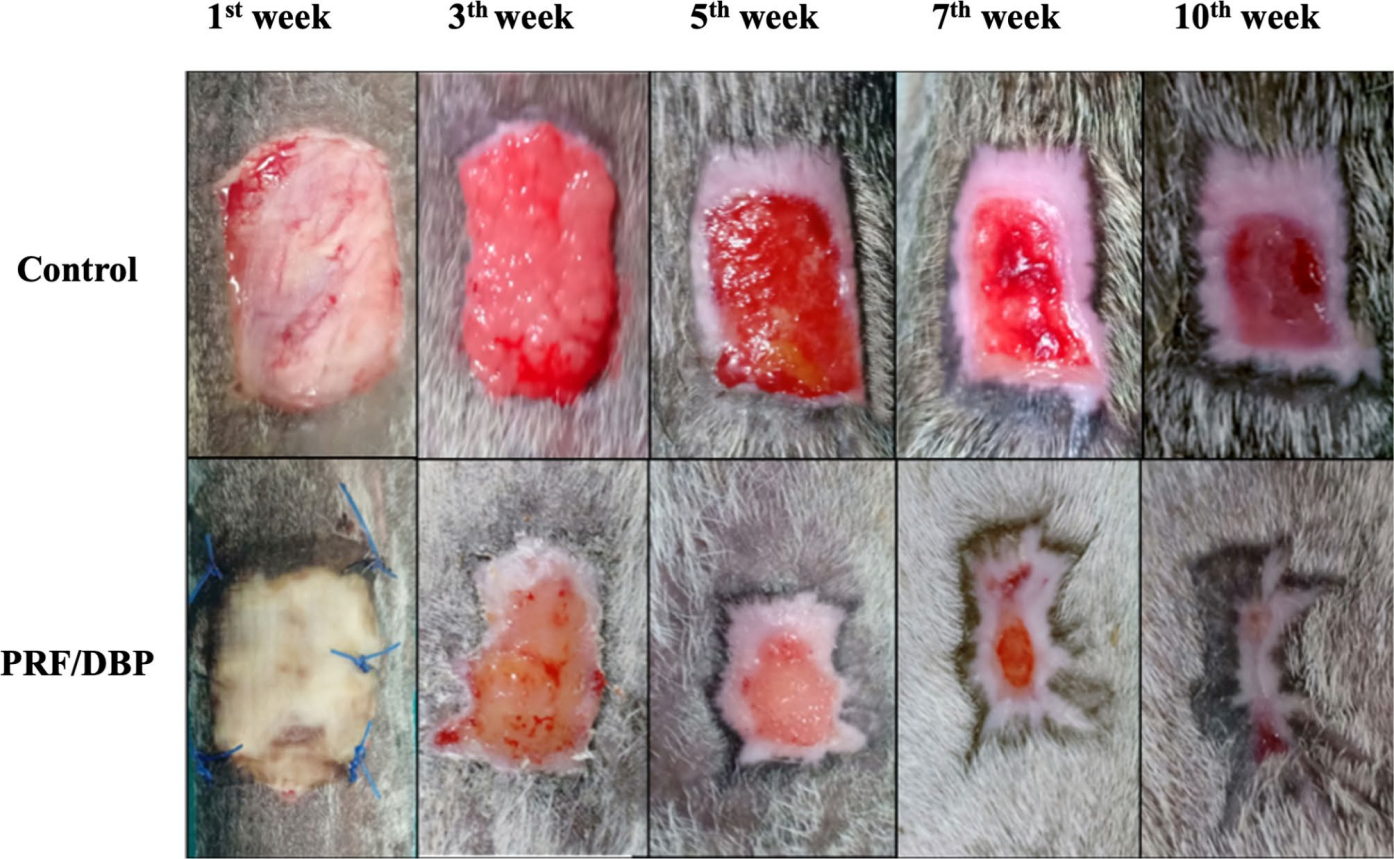
Progress of wound healing in control and different treatment wounds at different weeks

At the conclusion of the first week, granulation tissue was visible, and it was smooth, regular, pale pink, and under the skin’s surface. By the conclusion of the second week, the wounds were entirely filled with granulation tissues, and at the end of the third week, there was a slight elevation over the skin edge (Fig. 1).

The granulation tissue was hyperemic, with slight abnormalities. The swelling tissues were removed surgically from the incisions and bandaged until they healed. Three weeks after wound induction, there was a statistically significant difference in granulation tissue production score between control and PRF/DBP wounds (Table 1).

Epithelialization was initially seen at the third week post-wound induction and was evident by the fifth week; it continued to include all wound borders by the seventh week postoperatively. The wounds were progressively covered by epithelium (Fig. 1**&** Table 1).

In contrast, the bovine pericardium sheets in PRF/DBP-treated wounds were stable, well-fixed, and fully covered the wound beds one week after surgery. DBP sheets exhibited shrinkage and a glistening surface, although their wound-overlapping edges were colored black. The absence of inflammatory signs at surgical sites was observed (Fig. 1).

The exudate in all wounds was serous (watery, transparent, and in very small quantities) until it dissipated on the fifth postoperative day. All wound exudates have the benefit of a typical odor. The smoothness and uniformity of the granulation tissue, which was rosy red in color and seen in all wounds, were its defining characteristics (Table 1). In control wounds, wound boundaries exhibited modest edematous enlargement on the third day and light bleeding at the first bandage change. In addition, the animals exhibited a pain response, as shown by the mild finger pressure on the incision borders. On the sixth postoperative day, most wounds exhibited a significant reduction in edema. The skin’s sensitivity diminished progressively until it vanished at the end of the second week. In the first week, there was a discernible increase in wound contraction (10.2 ± 0.3 cm^2^), leading to retardation of wound contraction (-22.7 ± 4.6%; Table 1). The average time for wounds to heal was (86.3 ± 3.1 days).

At the end of the first week, wound diameters increased slightly (6.3 ± 0.06 cm2), inhibiting wound contraction. However, the wounds exhibited a minor degree of contraction during the second week, which was significantly identified by the third week (30.7 ± 2%). A significant degree of epithelization was also seen during the third week (34.6 1.8%). Five and seven weeks postoperatively, epithelization proceeded and expanded noticeably (69.7 ± 1.8; 83.6 ± 1.9, respectively). It was maintained in the tenth week after surgery until full recovery was reached (Table 1). The average time for wounds to heal was 61.3 ± 2.6 days.

### Histopathological results

**Fig. 2:**
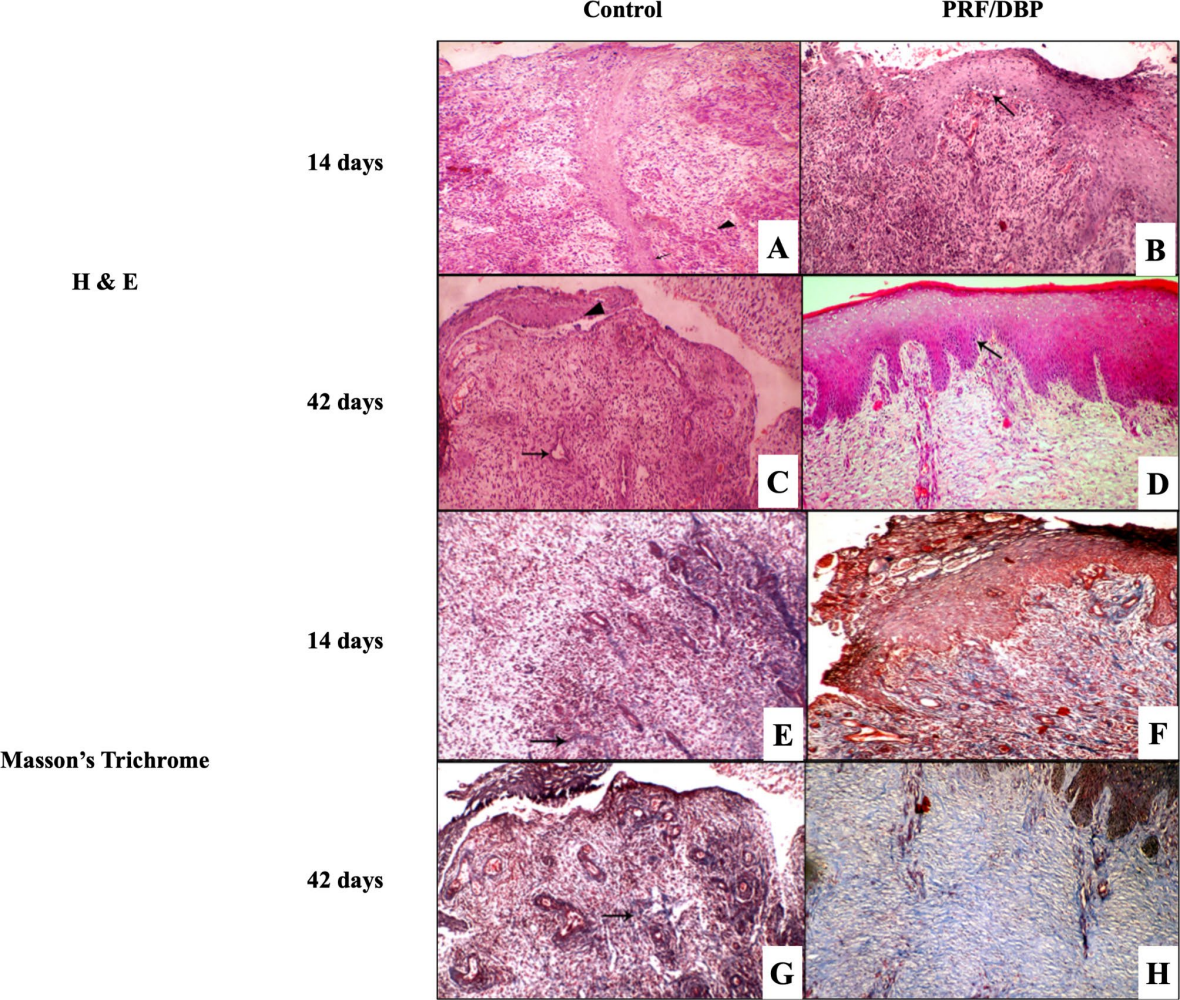
Histological findings of wound biopsies. **(A)** H&E staining of control wounds showed immature blood vessels (arrowhead), mild fibroplasia, and reepithelization limited to only epithelial stands with frequent mitosis in the basal cell layer (arrow) 14- days post wounds induction. **(B)** H&E staining of PRF/DBP wounds had an excess neutrophilic inflammatory infiltrate with fibroblastic proliferation. Reepithelization with the formation of bridges on the surface and formation of eosinophilic fibrin basement membrane (arrow) 14- days post-wound induction. **(C)** H&E staining of control wounds revealed fibroplasia with less collagenous deposition and some mature blood vessels (arrow), frequent mitosis in the basal cell layer in epithelium with the migration of epithelium on the surface of wound (arrowhead) 42- days post wounds induction. **(D)** H&E staining of PRF/DBP wounds showed complete layers of the epidermis, hyperplasia of rete ridges, and basal cell layer showing mitosis (arrow). Mature fibrous connective tissue and synthesized collagen bundles arranged in normal wavy bundles along the dermis (H&E, 100 xs) 42- days post wounds induction

Histological analysis of PRF/DBP treatment demonstrated the organization and formation of new collagen as well as some well-ordered dermo-epidermal cell interventions, such as leukocytic cells, neutrophils, macrophages, fibroblasts, keratinocytes, and the blood-forming-tissues endothelial cells. Masson’s trichrome staining; collagen fibers in PRF/DBP-treated wounds seemed compactly formed, well-organized, and diffused without segmentation, which was not observed in control wounds (Fig. 2). Semi-quantitative examination reveals that the PRF/DBP wound healing development is much greater than that of the control group. There were more dermo-epidermal cell interventions in the PRF/DBP-treated wounds than in the control wounds, including macrophages, neutrophils, fibroblasts, keratinocytes, endothelial cells, and leukocytic cells of the blood-forming tissue, besides the formation of newly organized collagen fibers (Table 2).

**Table 2:** Semi-quantitative evaluation of histological structures during skin wound healing in control and PRF/DBP treated groups in donkeys

Groups	Days	Epithelization	PMNL	Tissue macrophages	Fibroblasts	Neo-angiogenesis	New collagen
Control	14	0.4 ± 0.5^a^	0.6 ± 0.5^a^	0.4 ± 0.5^a^	0.6 ± 0.4^a^	0.7 ± 0.4^a^	0.3 ± 0.4^a^
PRF/DBP		2 ± 0.0^b^	2.7 ± 0.5^b^	2.2 ± 0.4^b^	2.5 ± 0.6^b^	2.7 ± 0.5^b^	2.3 ± 0.05^b^
Control	42	1.1 ± 0.4^a^	0.7 ± 0.4^a^	0.9 ± 0.0^a^	1.5 ± 0.5^a^	1.3 ± 0.5^a^	0.8 ± 0.5^a^
PRF/DBP		3 ± 0.0^b^	3 ± 0.0^b^	2.8 ± 0.4^b^	2.8 ± 0.4^b^	3 ± 0.0^b^	3 ± 0.0^b^

Means with different superscript letters in the same column at the same time point are significantly different at p < 0.05

## Immunohistochemical results

**Fig. 3:**
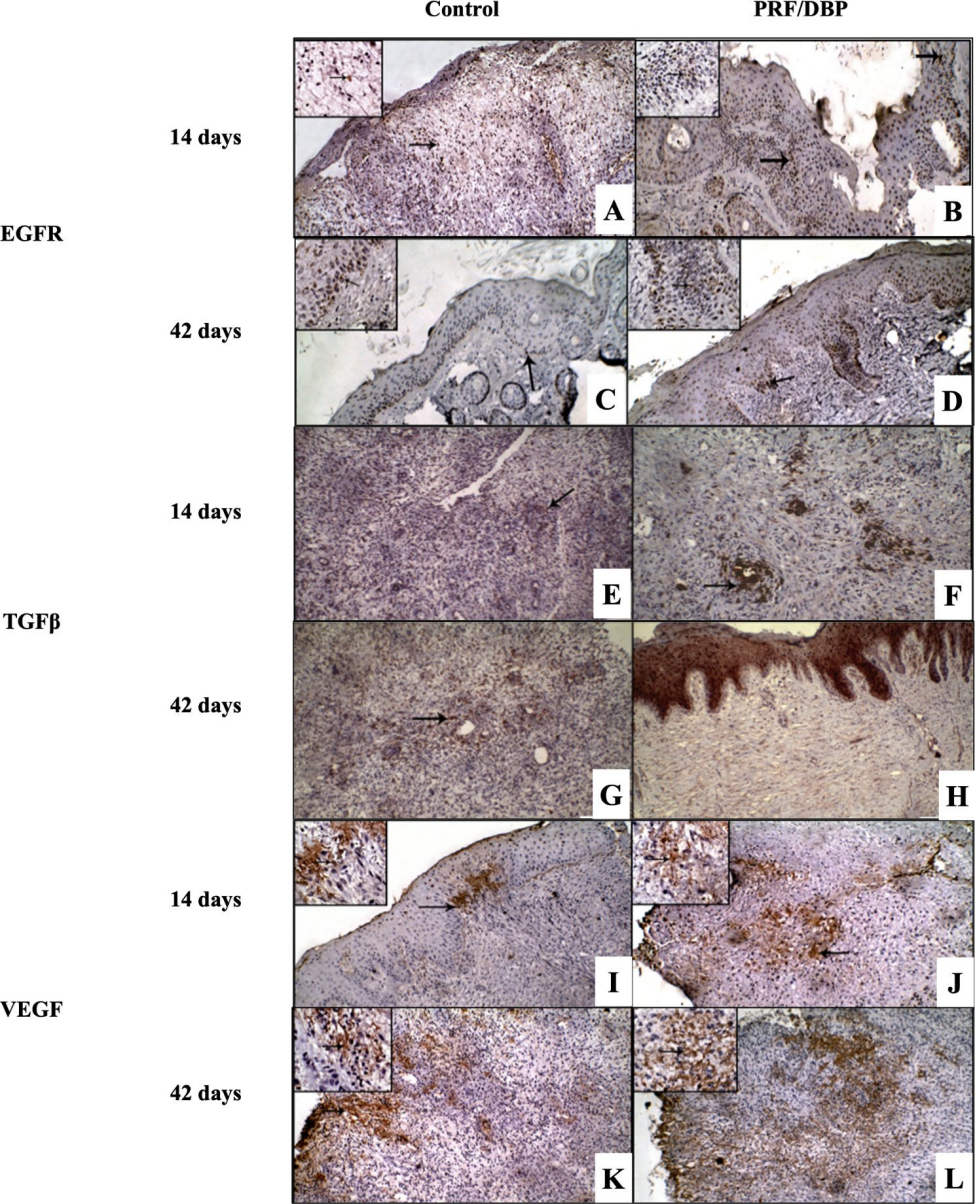
Immunohistochemical staining, DAB immunostaining, hematoxylin as a counter stain, 100 x. **(A)** EGFR in control wounds showed a mild positive brown immunostaining limited to the superficial of the wounds (arrows) 14- days post wounds induction. **(B)** EGFR in PRF/DBP wounds is a strong positive in basal cell layers and stratum corneum (arrows) 14- days post wounds induction. **(C)** EGFR in PRF/DBP wounds a mild positive brown immunostaining for epithelial cells in basal cell layers (arrows) 42- days post wounds induction. **(D)** EGFR in control wounds a strong positive staining for epithelial cells at stratum corneum and stratum basalis (arrows) 42- days post wounds induction. **(E)** TGFβ in control wounds showed positive faint brown immunostaining for fibroblasts 14- days post wounds induction. Newly formed fibroblasts (arrows). **(F)** TGFβ in PRF/DBP wounds showed moderate positive brown immunostaining for fibroblasts 14- days post wounds induction. Newly formed fibroblasts (arrows). **(G)** TGFβ in control wounds showed mild positive brown immunostaining for fibroblasts 42- days post wounds induction. Newly formed fibroblasts (arrows). **(H)** TGFβ in PRF/DBP wounds showed strong positive brown immunostaining for fibroblasts 42- days post wounds induction. **(I)** VEGF in control wounds showed positive faint brown immunostaining for fibroblasts 14- days post wounds induction. Newly formed angioblasts (arrows). **(J)** VEGF in PRF/DBP wounds showed moderate positive brown immunostaining for fibroblasts 14- days post wounds induction. Newly formed angioblasts (arrows). **(K)** VEGF in control wounds showed mild positive brown immunostaining for fibroblasts 42- days post wounds induction. **(L)** VEGF in PRF/DBP wounds showed strong positive brown immunostaining for fibroblasts 42- days post wounds induction

A qualitative immunohistochemical examination of PRF/DBP and control wounds revealed a considerable difference in immunostaining for EGFR, TGFβ, and VEGF and between control and PRF/DBP wounds. At 14- and 42-days following wounds induction, the anti-EGFR antibody immunohistochemical examination was substantially elevated and more intense staining in PRF/DBP than in control wounds (302.3 ± 5.2 and 420.3 ± 10.8 versus 48.8 ± 1.7 and 80 ± 3). In contrast, VEGF in PRF/DBP -treated wound tissue was substantially increased and considerably improved than the control (316.3 ± 4.6 and 495.7 ± 6.4 versus 138 ± 2 and 161 ± 5.1). The mean cell number immunostained for TGFβ appeared to be a marked improvement in the PRF/DBP-treated wounds compared with the control (175 ± 4.8 and 222.6 ± 16.2 versus 35.5 ± 3.2 and 90 ± 2.1; Fig. 3).

### Gene expression results

At 14 days following induction of wounds, COL3α1, FGF-7, VEGF-A, and TGFβ1 expression was substantially elevated in the PRF/DBP-treated wounds (P ˂ 0.0001) than controls (6.7 ± 0.15, 8.06 ± 0.13, 6.72 ± 0.14, and 7.05 ± 0.14 versus 1 ± 0.03, 1 ± 0.05, 1 ± 0.09, and 1 ± 0.02 folds, sequentially). Furthermore, at 42 days following induction of wounds, a relative expression of the aforementioned genes exhibited considerably regulated PRF/DBP-treated wounds (P ˂ 0.0001) compared with the control (11.3 ± 0.18, 13.93 ± 0.08, 12.55 ± 0.11, and 12.25 ± 0.15 versus 1 ± 0.1, 1 ± 0.3, 1 ± 0.04, and 1 ± 0.2 folds, sequentially).

## Discussion

To our knowledge, this study was the first one that discussed the impact of topical dressing of a PRF/DBP combination in treating donkeys’ cutaneous distal limb wounds. Poor prognosis, unsatisfactory cosmetics, prolonged wound healing, and in horses’ lower extremities lead to elevated risks of the increased motion wound area, lack of soft tissue support, lack of blood supply, as well as bacterial and infection contamination. Consequently, the wounds in the lower extremity are considered crippling as well as life-threatening to horses [22].

Wounds in equine distal limbs continue to be a significant challenge for veterinarians, as they are typically healed by secondary intention, which is prone to complications because of the slower healing rate compared to other body wounds, delayed epithelialization, formation of massive granulation tissues, elevated skin retraction, and wound contraction. Therefore, our study’s primary objective for managing wounds in distal limbs in equines was to increase likelihood of a return to full athletic performance, accelerate epithelialization, contraction rate, and ultimate cosmetic attendance, as well as diminish scar tissue and exuberant granulation tissue [3].

Using PRF to maintain growth factors active, preserve them from proteolysis for a comparatively longer duration (approximately 14 days), and efficiently induce tissue regeneration was shown in the present work [23]. In addition, PRF offers a unique kind of thick fibrin biomaterial that permits the gradual release of several growth factors. Therefore, it is regarded as a crucial biomaterial for surgical problems in horse species, and it offers the most native provisional matrix feasible for wounds [24, 25]. Moreover, obtaining autologous PRF is a simple, easy, and economical method and successful PRF production is dependent on quick transfer for centrifugation within one minute as well as blood collection [6, 7].

DBP, on the other contrary, is a xenograft, and it is vital to decellularize BP because it contains a robust antigen that induces immunological responses in the recipient, resulting in graft rejection. Moreover, decellularization attempts to eliminate the cellular component from tissues while organs as well as their extracellular matrix components, maintain all of the required signals for cell preservation [26]. Additionally, decellularization of BP avoids immunological rejection and reduces inflammatory response after wound administration, as illustrated by [14, 27]. Prior to DBP fragment fixation above PRF, it was cut 0.5 cm2 larger than the wound to retain the wound’s moist environment as much as possible and washed in saline, then penicillin antibiotic solution for 15 min to rehydrate and raise the wound’s resistance to infection, as confirmed by [28].

DBP sheets’ mechanical cleaning utilizing dry gauze was manually done to dissect all connective tissues and undesirable fat from the pericardium as well as to create a rough surface. The roughness of the biomaterial regulates tissue biological response to the implants and plays a vital role in cellular behavior and adhesion [29, 30].

Compared to previous research that done in equine [8, 10], wound healing following PRF/DBP dressing had a shorter interval than DBP or PRF alone; it was (61.3 ± 2.6 versus 71.6 ± 3.8 and 69.5 ± 1.6, respectively) days following induction of wounds. It was observed that the potential augmentation impact of PRF/DBP combination in managing donkeys’ lower limbs.

The absence of immunological rejection and inflammatory signs was detected when the wound was treated with PRF/DBP, which was ascribed to the capacity of PRF to inhibit the inflammatory cascades, the autologous synthesis of PRF, and the removal of non-collagen components (decellularization) from the BP [27, 31, 32]. Relative findings were reported by [31], who examined the DBP impact as a dural graft material in human patients scheduled for a spinal surgery [8, 10, 14], who assessed the impact of the acellular bovine pericardium on cutaneous wound healing in rabbits as well as PRF in donkeys.

In the current study, to prevent fog wound healing assessment in the present investigation, a single dose of systemic antibiotic was administered without NSAID [33]. There is no agreed-upon paradigm for the use of anti-inflammatory medications; [34] suggested the utilization of painkillers such as butorphanol in donkeys postoperatively to eliminate complications like delayed wound healing or prolonged recovery time.

Our findings suggest that when DBP was utilized to cover a wound, it may have imitated the beneficial features of a scab, namely wound protection and preservation of a healing-friendly environment. According to [35], a scab is capable of stopping bleeding, protecting the wound, inhibiting bacterial infections, and providing a framework for tissue regeneration. Moreover, temperature, mobility limitation, and the presence of a moist healing environment are likely key contributors to wound healing.

In addition to the growth factors sequestered in the PRF matrix, the shrinking and black color of DBP resulting from ECM degradation plays a vital role in the healing process via the release of growth factors sequestered inside the matrix [36]. As a result, the cells around the wound proliferate, disseminate throughout the wound cavity, and preserve the integrity of the tissue. In addition, [27] reported that host cells degraded the scaffold, resulting in the development of site-specific functional host tissues.

In the current research, distal limb wounds increased first and subsequently shrunk as a result of higher mobility and the skin tension forces retracting the skin boundaries and increasing wound expansion, as indicated by [37].

It was discovered that the balance between collagen production and degradation, particularly in the distal side of distal limb wounds, was essential for preventing excessive granulation tissue and delaying wound healing. Furthermore, PRF/DBP wounds showed quicker healing rates than control wounds, indicating that DBP retains the ECM, enhances growth factor release, and induces angiogenesis. These results are consistent with those of [38], who reported that the processing method impacts the porcine dermal ECM. Therefore, proper processing procedures must be carefully chosen to preserve ECM’s positive effects in biological scaffolds [39].

The wounds treated with PRF/DBP exhibited an earlier onset and a greater rate of epithelialization than the control wounds. This finding may be attributed to the keratinocyte growth factor, a significant growth factor that promotes wound epithelialization and keratinocyte differentiation into a variety of epithelial cell types, as stated by [36].

In our research, secondary intention wound closure was accomplished by epithelialization and wound contraction. Significantly greater wound contraction in PRF/DBP-treated wounds compared to control wounds may be because of the presence and activity of myofibroblasts, which are responsible for the centripetal motion of the wound edges as well as an elevated deposition of fibroblast infiltration and fibrous connective tissues. The same finding was reported by [40]. Furthermore, we discovered that fixing DBP sheets to the wound borders did not inhibit contraction but may inhibit expansion, which is compatible with [28], who demonstrated a favorable impact of the pericardium on granulation tissue in horses with distal limb wounds treated with pericardium dressing.

Histopathological analysis of PRF/DBP-treated wounds revealed significant proliferative activity in the skin layers. Enhanced epithelialization was a response to the extensive mitotic activity of proliferative keratinocytes, which resulted in the formation of distinctive rete ridges by interdigitating between well-structured connective tissues and hyperplastic neo-epidermis in the dermis layer. These results concur with those of [41], who reported that PRF improved epithelization in gingival wound healing. Furthermore, the histological results revealed the integration of scaffolds with the host tissue, which had been eliminated throughout the course of the study in contrast to untreated ones. The ECM sheet interacted with the wounds and offered protection, adhesion, and a wet healing environment. These findings supported the advantages of human placenta-derived ECM, including bioactive compounds, on full-thickness skin wound healing in a rat model [39]. In addition, a robust proliferative effect was seen in both the dermal and epidermal layers. SDF-1 and TGF β 1 were responsible for the increased mitotic activity of proliferative keratinocytes, which resulted in increased epidermal epithelialization [42].

VEGF’s cell quantification, a potent angiogenic factor generated by a range of cell types, including fibroblasts, mast cells, macrophages, endothelial cells, and keratinocytes [43], was considerably greater in wounds treated with PRF/DBP. This result implies that PRF and DBP represent a constant source of VEGF, which substantially helps to heal. The occlusive aspect of the wound dressing promotes healing and preserves the wound’s moisture [44]. Moreover, new capillary formation across newly created granulation tissues stimulated the healing process, comparable with reported observations in rats and humans [27].

In this study, VEGF’s immunohistochemistry substantially increased PRF/DBP-treated wounds compared to untreated wounds. Quantification of cells immunostained for VEGF revealed the vital role of both DBP and PRF as an essential source of VEGF that has a vital role in healing damaged tissues, as demonstrated by [45, 46].

Likewise, EGFR immunohistochemistry considerably increased PRF/DBP-treated wounds relative to untreated wounds. The quantity of EGFR-immunostained cells was greater in treated wounds due to the amount of EGF administered to the wounds. EGF is secreted by platelets and is appealing to fibroblasts; its topical administration promotes wounds’ epidermal regeneration as well as tensile strength [47]. In addition, the increased amount revealed that DBP plays a critical function in boosting the epithelization process, which is essential for healing. These results indicated that the scaffolds were effectively incorporated into wounds. The closure was accomplished via re-epithelialization by keratinocytes in endogenous wounds [48] since EGFR significantly enhances the rate of macroscopic healing in PRP-treated tissue compared to control wounds.

In addition, TGFβ expression by immunohistochemistry showed a significant increase in PRF/DBP treated wounds compared to control ones. This result could be attributed to PRF being a source of TGF in fibroblasts activation for pre-collagen creation that stimulates the deposition of collagen, and the process of wound repair and DBP was a source of TGF in promoting epithelial cell proliferation, angiogenesis, and the organization of wound sites, which aligns with [49].

Donkeys’ gene expression in this study exposed to PRF/DBP applied to distal limb wounds revealed considerably elevated gene expressions and FGF-7, VEGFA, TGFβ1, and COL3α1 at all time points compared to controls. Similar findings were reported by [50, 51] that can be attributable to the number of growth factors provided by both PRF/DBP dressings. These results agree with [25], who examined the impact of platelet-rich plasm as well as its growth factors on equine distal limb wounds. Furthermore, the higher expression revealed PRF’s contribution that provides a steady release of growth factors for a 10–14 days interval in fibroblast activation for pre-collagen formation prompting collagen deposition and healing wound.

## Conclusion

Using PRF combined with DBP has resulted in numerous advantages, including the maintenance of a better moist environment required for the healing process, the slowing of the degradation process of the DBP sheet, and the preservation of the sheet’s viability as much as possible, allowing the wounds to retain the large benefits from the quantity and duration of the growth factors. Moreover, minimizing the wounds’ exposure to iatrogenic and environmental factors. The combination of PRF and DBP could be considered a good biological scaffold for donkey distal skin limb wounds. It is a safe, economical, and efficient treatment for cutaneous distal limb wounds in donkeys.

## Materials and methods

### Animals

G-Power software, version 3.1.9.7, was utilized for determining the sample size based on 80% statistical power and 5% type I error (alpha). It was found that N = 12 is the adequate sample size to test the study hypotheses.

Twelve clinically healthy male donkeys, with ages ranging from 5 ± 2.1 years and weight of 180 ± 30 kg, with no scars or blemishes on the metacarpal regions, without lameness or pain reaction, were included in this study.

These donkeys were purchased from The Animal House of the veterinary teaching hospital, Faculty of Veterinary Medicine, Mansoura University, Dakahlia province (Egypt). Equiveen Paste (0.2 mg/kg Per Os; Ivermectin Paste, Adwia Company, Egypt) as an anthelmintic drug was administered to donkeys fourteen days before the study. Animals were kept in separate stables as well as received a balanced diet. After the end of the study donkys were returned to the Animal House of the veterinary teaching hospital, Faculty of Veterinary Medicine, Mansoura University.

The Animal Welfare and Ethics Committee of the Faculty of Veterinary Medicine validated all animal care and testing procedures following the Guidelines for Animal Use and Care published by the Faculty of Veterinary Medicine, Mansoura University, Egypt. All methods were reported in accordance with ARRIVE guidelines.

### Platelet-rich fibrin (PRF) preparation

**Fig. 4:**
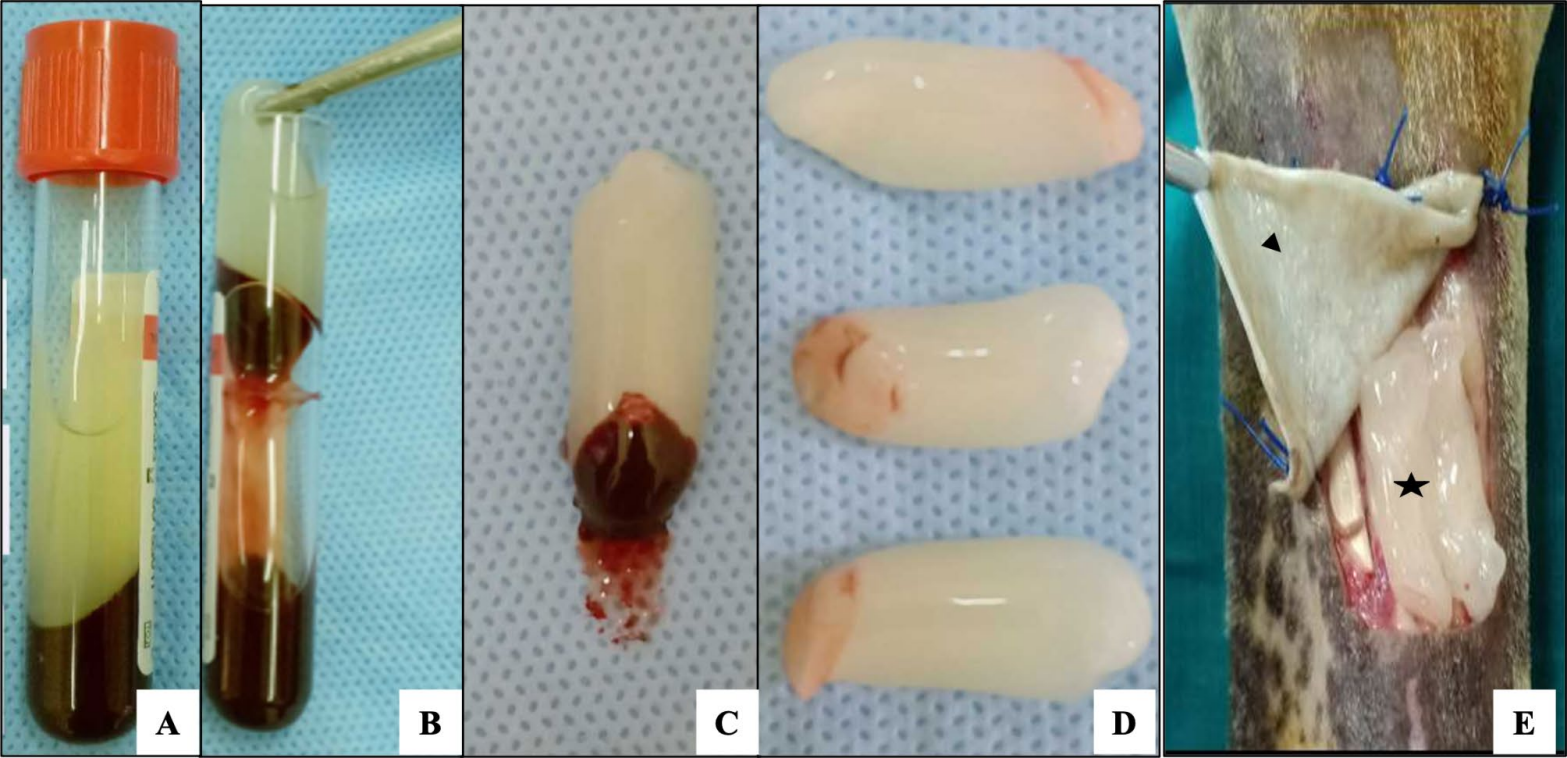
PRF preparation and application; (**A)** A centrifuged blood sample, **(B)** Catching of the PRF clot with tissue forceps, **(C)** PRF clot adhered to RBCs, **(D)** PRF clot after removal of RBCs and **(E)** Topical application of both PRF/DBP on wounds; star pointed to PRF and head arrow pointed to DPB.

According to (Dohan et al. 2006), 20 ml blood samples were withdrawn from each donkey before being immediately subdivided into five sterile vacutainer tubes ( each containing 4 ml without anticoagulant). Centrifugation of tubes was immediately performed at 3000 rpm/10 min. Three separate layers were produced: a packed red cell layer at the bottom, a solid fibrin clot (middle layer), and a straw-colored acellular plasma layer on the top. The top straw-colored layer was discarded, and the middle layer was collected. Utilizing sterile tissue forceps to remove the PRF clot, sterile scissors separated PRF from the RBC base and then placed it on sterile gauze (Fig. 4A-D).

### Decellularized bovine pericardium (DBP) sheet preparation

The bovine pericardium was taken from the local slaughterhouse immediately following bovine slaughtering. In order to transfer the bovine pericardium to the laboratory, it was immersed in phosphate-buffered saline (PBS; PH = 7.2). In order to remove the adhered blood, tissues were rinsed gently with PBS. Utilizing dry gauze, mechanical cleaning was manually carried out to eliminate all connective tissues and undesired fat from the pericardium. Decellularization of the pericardium was done utilizing a combination of ethanol combination (4%) and peracetic acid (0.1%) for two hours and cleaned with deionized water and PBS for 15 min. At 4 °C, the prepared DBP was stored in PBS with Gentamycin (1%) (Gentacure-10, Pharma Swede Co, Egypt) at 9:1 volume respectively till usage [52].

### Experimental design

Pre-anesthetic data, including respiratory rate, breed, age, body weight, lung sounds, mucous membrane color, heart rate, cecum sound and mobility rate, skin hydration test, capillary refill time, and body temperature, were recorded in all donkeys. Prior to anesthesia induction, food was withdrawn for 10 h. A 14-gauge (160 mm catheter) was applied in the jugular vein under local anesthesia with mepivacaine 2%.

Prior to anesthesia induction, the donkeys were IM injected with 4.5 mg/kg bodyweight benzathine penicillin containing 1ml per 25 kg bodyweight: Norocillin LA, Norbrook Co.UK) as well as a penicillin-based antibiotic dosage (6 mg/kg bodyweight procaine penicillin. Thirty minutes before induction of anesthesia, donkeys were IV administrated of of 0.05 mg/kg acepromazine maleate (Castran, 1.5%; Interchemie Co., Holland). Donkeys were IV sedated with 1 mg/kg xylazine HCL (Xylaject; 2%; Adwia Company., Egypt). Five-minute post sedation, induction of anesthesia was done via IV administration of 2.2 mg/kg ketamine (Narketan 10 ad us. vet.; Chassot AG, Belp-Bern, Switzerland 10 mg/ml), along with 0.02 mg/kg diazepam (Valium 10 mg/ml; Roche Pharma Schweiz AG, Reinach, Switzerland). Then anesthesia was maintained by a continuous infusion rate of 0.2 mg/kg/min propofol (Pofol, 1%; Eimc Co., Dongkook, Korea). All animals were in dorsal recumbency with their forelimbs completely extended. Aseptic preparation of the metacarpal region by circumferentially clipping and shaving of hair was followed by Betadine disinfection in preparation for induction of aseptic wounds. A tourniquet was applied above the elbow joint. A sterile metal square template (3 × 3 cm^2^) was placed at the dorsolateral side and in the mid of the metacarpal region. A No. 22 scalpel blade was used to create full-thickness wounds, and a surgical incision was made around the square metal template. Subsequently, mechanical pressure and tampons were used to control hemostasis on both forelimbs of each donkey (12 defects per group). In addition, control and PRF/DBP wounds were done on each donkey’s left and right limbs, respectively. Utilizing polypropylene monofilament suture material, PRF/DBP wounds were filled with PRF clot and covered with DBP sheets fixed by simple interrupted suture patterns (Prolene, Ethicon, Inc., Somerville, N.J.; Fig. 4E). The application of protective non-adherent dressings was made to the two wounds of the forelimb, and the defects were left to heal spontaneously. Three layers of a standard non-adherent distal limb bandage were utilized for wrapping the wounds. The bandage’s first inner contact layer (Derma-Tulle, Gauze Pads, Telfa, 10*15, Dressing Medical Me, Egypt) was non-adherent dressing sterile gauze. The second padding layer (Surgical pad 10ҳ10cm, Tri M Medical, 10th of Ramadan City, Cairo, Egypt) was a sterile absorbent dressing pad (Sofpad El Mahalla Co., El-Mahalla, Egypt) secured with soft, elastic roll cotton. The third layer was an elastic adhesive tape (Silk Plast Adhesive Tape 10 cm, Pharmaplast Co, Kafr El-Zayat, Egypt) as well as gauze (10 cm, El Mahalla Co., El-Mahalla, Egypt). The tourniquet was removed at the end of surgery and after applying a bandage.

### Postoperative medication

Donkeys were put in a padded recovery room and received 50 µg/kg of butorphanol (Torbugesic, Fort Dodge, IA, USA) for three successive days [34]. Penicillin-based antibiotic ( single dosage, 6 mg/kg, Norocillin LA, Norbrook Company, United Kingdom) was intramuscularly injected into all animals. Eight-hour post-recovery were allowed to eat and drink.

The control wounds were washed with normal saline before replacing bandages at the 4th, 7th, 10th, 13th, 16th, 19th, 21st, 24th, and 28th days post-wound induction. Otherwise, in the FRP/DBP wounds, donkeys were kept in a standing position by IV injection of 1.1 mg/kg xylazine and under the effect of local mepivacaine HCl infiltration (Mepecaine, 2 mg/ml, Alexandria Co; Egypt) before replacing the FRP/DBP dressing at 7th, 14th, and 21st days postoperatively.

### Wound healing evaluation

#### Clinical and macroscopical evaluation

A single-blinded investigator (M.A.) evaluated and monitored the wounds. By using the digital caliper, measurements of wound sizes were taken, and by utilizing the digital photographs, the wound contraction, epithelialization, as well as formation of granulation tissue were monitored immediately following wound induction till full epithelialization of wounds at weekes number one, three, five, seven, nine, and following the induction of wounds.

The percentages of epithelialization, wound contraction, and healing were determined based on the subsequent formula [53]; wound contraction% = 100 - (wound size at day (x) mm^2^ / wound size at day (0) mm^2^ × 100).

Wound epithelialization%= Size of epithelialization area at day (x) mm^2^ / size of the wound at day (0) mm^2^ × 100. While the wound healing%= 100 – (granulation tissue at day (x) mm^2^ / size of the wound at day (0) mm^2^ × 100).

The formation of granulation tissues was scored based on [54]. Surgical excision of exuberant granulation tissue (i.e., grade 4) was done to the surrounding epithelium level under the general anesthesia illustrated above. When wounds were completely covered by epithelium, they were regarded to have healed.

### Histopathological evaluation

Tissue biopsies were taken on days 14 and 42 following the first treatment under the effect of general anesthesia mentioned above. The collection of samples was done under aseptic conditions from wound edges with 2 to 3 mm of normal skin. The samples were preserved for 24 h in 10-percent neutral-buffered formalin before being embedded in paraffin, divided into (5) microns on a rotary microtome, then stained with hematoxylin and eosin (H&E) to assess morphological characteristics of Masson Trichrome and tissues for highlighting fibrous connective tissues throughout the remodeling and proliferation stages of wound healing. Histopathological examination factors included the degree of collagen maturity, neovascularization, fibroblast cell presence, fibroplasia process, granulation tissue formation, and inflammation. The histological sections were scaled and semi-quantitative analyses according to [55].

### Immunohistochemical analysis evaluation

Tissues biopsy immersed in paraffin were sectioned at a thickness of 4 μm as well as placed on saline-coated glass slides before deparaffinization in dehydrated and xylol in varying concentrations of ethanol. At a pH of 6.0, extraction of antigen was performed by 10-minute autoclaving at 120 ^o^C. Endogenous peroxidase activity was inhibited for 10 min with 3% H_2_O_2_. Subsequently, treatment of tissue slices was done utilizing primary antibodies against TGFβ, FGF, and EGFR (ready to use, Bio Genex). Tissue slices were incubated for one hour at an ambient temperature and then washed three times utilizing phosphate buffer saline. Following, 30 min at ambient temperature utilizing anti-rabbit secondary antibodies before visualization with the three diaminobenzidine tetrahydrochloride liquid system (Dako) for 5 min at ambient temperature. Finally, hematoxylin was used to counterstain tissue slices.

### Gene expression analysis

Trizol reagent was used to homogenize and lyse tissue samples (Invitrogen, Carlsbad, CA, U.S.A.). Utilizing an Implen spectrophotometer (Implen, Westlake Village, CA, U.S.A.), examination of RNA concentrations and purities were examined. Production of cDNA was done using 1 g of total RNA per sample as well as a Sensi Fast cDNA synthesis kit (Bioline, Taunton, MA, U.S.A.). The newly synthesized cDNA was blended with a master mix (TaKaRa, Otsu, Japan) and relevant target primers to examine the response of tissues to the induced wound. The transform growth factor β1 (TGFβ1) to was utilized for assessing wound closure, collagen-type 3 α1 (COL3α1) for evaluating collagen deposition, fibroblast growth factor 7 (FGF-7) for evaluating the re-epithelization of wounds, and vascular endothelial growth factor A (VEGF-A) for evaluating angiogenesis. Processes were performed utilizing a Pikoreal system (Thermo Fischer Scientific, Waltham, MA, U.S.A.). The excised tissues’ gene expression was compared to the housekeeping gene glyceraldehyde 3-phosphate dehydrogenase (GAPDH) at each time point (Table 3).

**Table 3:** List of primers used in gene expression analysis

Gene	Primer sequence	Accession number
**GAPDH**	F: GGAGTAAACGGATTTGGCC	XM_014834961
	R:CATGGGTGGAATCATACTGAAA	
**TGF-β1**	F:TAATTCCTGGCGCTACCTCA	HM569606
	R:CATGAGGAGCAGGAAGGGT	
**FGF-7**	F: GACAGTGGCAGTTGGAATTGT	NM_001163883
	R: CAACAAACATTTCTCCTCCACTG	
**VEGF**	F: TCATTTCTCCAGGGTTTACCCT	XM_014837457
	R:ATTTGGGGGAGTAGAAGAGCAA	
**COL3 α1**	F: TTCCTGGGAGAAATGGTGACC	XM_014852914
	R:GGAGAATAGTTCTGACCACCAGT	

The results were normalized according to the level of GAPDH. There were three replicates of each biological sample, and the data were reported as standard deviations and means.

### Data analysis

All statistical analyses were performed utilizing the 21st version of the SPSS software (IBM Inc, Chicago, IL). Data normality was tested using the Kolmogorov-Smirnov test. In addition, the non-parametric Kruskal–Wallis test with the post-hoc Dunn’s multiple comparison test were applied at varying time points to determine the statistical differences between examined parameters. The two-way repeated measures ANOVA was utilized for the parametric data, whereas Wilks’ lambda test was utilized to examine time x treatment binding evidence and within-group. Additionally, Wilks’ lambda test indicated statistically substantial differences between groups. A one-way ANOVA was performed at each time point when there was a significant effect. All data were expressed as the mean ± standard deviation (SD), whereas the results of the gene expression analyses were expressed as the mean with the standard error of the mean (P-value 0.05).

## Data Availability

This article contains all data generated or analyzed throughout the course of this research.
